# Effects of 5-Aminolevulinic Acid Supplementation on Gas Production, Fermentation Characteristics, and Bacterial Community Profiles In Vitro

**DOI:** 10.3390/microorganisms12091867

**Published:** 2024-09-09

**Authors:** Zhenkai Hao, Zhuangzhuang Guo, Ning Zhang, Jing Wang, Jiabao Xu, Weiyu Zhang, Qiang Liu, Cong Wang, Yawei Zhang, Yuanqing Zhang

**Affiliations:** College of Animal Science, Shanxi Agricultural University, Taiyuan 030031, China

**Keywords:** 5-ALA, beef cattle, in vitro rumen fermentation, bacterial diversity

## Abstract

To investigate the effect of 5-aminolevulinic acid (5-ALA) on in vitro rumen gas production, fermentation characteristics, and bacterial community profiles, five levels of 5-ALA (0, 100, 500, 1000, and 5000 mg/kg DM) were supplemented into a total mixed ration (concentrate/forage = 40:60) as substrate in an in vitro experiment. Results showed that as the supplementation level of 5-ALA increased, asymptotic gas production (b) decreased linearly and quadratically (*p* < 0.01) while the dry matter degradation rate increased quadratically (*p* < 0.01). Meanwhile, the propionate concentration of 72 h incubation fluid increased linearly (*p* = 0.03) and pH value increased linearly and quadratically (*p* < 0.01), while the concentrations of butyrate, isobutyrate, valerate, isovalerate, and NH_3_-N and the ratio of acetate/propionate (A/P) decreased linearly and quadratically (*p* < 0.05). There was no significant difference in any alpha diversity indices of bacterial communities among the various 5-ALA levels (*p* < 0.05). PCoA and PERMANOVA analysis revealed that the bacterial profiles showed a statistical difference between the treatment 5-ALA at 1000 mg/kg DM and the other levels except for 5000 mg/kg DM (*p* < 0.05). Taxonomic classification revealed a total of 18 and 173 bacterial taxa at the phylum and genus level with relative abundances higher than 0.01% in at least half of the samples, respectively. LEfse analysis revealed that 19 bacterial taxa were affected by 5-ALA levels. Correlation analysis showed that *Actinobacteriota* was positively correlated with the gas production parameter b, the ratio of A/P, and the concentration of butyrate, isovalerate, and NH_3_-N (*p* < 0.05) and negatively correlated with pH (*p* < 0.05). *WPS-2* exhibited a negative correlation with the gas production parameter b, the ratio of A/P, and the concentration of butyrate, valerate, isobutyrate, isovalerate, and NH_3_-N (*p* < 0.05), along with a weaker positive correlation with pH (*p* = 0.04). The *Bacteroidales BS11 gut group* was negatively correlated with the concentration of propionate but positively correlated with gas production parameter b and the concentration of butyrate and NH_3_-N (*p* < 0.05). The *Lachnospiraceae NK3A20 group* was found to have a positive correlation with gas production parameter b, the ratio of A/P, and the concentration of butyrate, isobutyrate, isovalerate, valerate, total VFA, and NH_3_-N (*p* < 0.05), but a highly negative correlation with pH (*p* < 0.01). Differential metabolic pathways analysis suggested that metabolic pathways related to crude protein utilization, such as L-glutamate degradation VIII (to propanoate), L-tryptophan degradation IX, and urea cycle, increased with 5-ALA levels. In summary, including 5-ALA in the diet might improve energy and protein utilization by reducing the abundance of *Actinobacteriota*, the *Bacteroidales BS11 gut group*, the *Lachnospiraceae NK3A20 group*, and certain pathogenic bacteria and increasing the abundance of WPS-2.

## 1. Introduction

Over the past century, antibiotics have been widely used as animal feed additives for their excellent disease-prevention and growth-promoting effects. In recent years, however, it has become increasingly apparent that antibiotic abuse in animal feed can lead to many serious issues, such as the development of superbugs, reduced immunity in livestock, and environmental contamination [1]. Many countries have explicitly banned the use of antibiotics in animal feed, which is likely to impact livestock production. Therefore, a variety of novel compounds have been studied as potential replacements for antibiotics [2].

5-ALA is a non-protein-synthesized endogenous amino acid in almost all organisms, including prokaryotes and eukaryotes. It is produced in animals and fungi through the C4 pathway, which involves the condensation of glycine and succinyl-CoA. Plants, bacteria, and archaea produce it through the C5 pathway, which involves the use of glutamate [3]. 5-ALA can chelate with different metal ions, such as Fe^2+^, Mg^2+^, Co^2+^, and Ni^2+^, to produce different tetrapyrrole compounds, including heme, chlorophyll, vitamin B_12_, and cofactor F430 [4]. The utilization of heme by rumen microbiota as a source of iron or heme may affect the activity of rumen fiber-degrading microorganisms (particularly anaerobic fungi) [5], which may further affect the degradation of dietary crude fiber in rumen. Cobalamin (vitamin B_12_) is a key cofactor in liver gluconeogenesis and one-carbon metabolism [6], which impacts ruminants’ productivity. In addition, Cofactor F430 plays a crucial role in methanogenesis by rumen microorganism [7].

It has been reported that 5-aminolevulinic acid, also known as 5-ALA, may enhance humoral immunity while minimizing immune stress as an immunomodulator, making it a potential alternative to antibiotics for animal disease prevention [8,9]. Including 5-ALA in the diet of weanling pigs has been found to increase the levels of differentiation antigen-positive cells^2+^ (CD^2+^), CD^8+^, B cells, and major histocompatibility complex I (MHC-I) and II (MHC-II) in the blood, which can enhance their ability to resist disease [10], and reduce tumor necrosis factor-α favoring the immune response during inflammation [11]. Improvements in immune status have also been found in broiler chickens and cows [8,9]. It has been reported that incorporating 5-ALA in the sow diet leads to an improved effect on the gut microbiome [12]. Furthermore, the supplementation of 5-ALA has been found to enhance the average daily growth (ADG), feed efficiency (F/G), and dry matter (DM) digestibility of weaned piglets [13]. It is also worth noting that the increase in albumin and casein contents in milk suggests that 5-ALA may improve the efficiency of dietary protein utilization [8].

Most tetrapyrrole compounds, derivatives of 5-ALA, can be synthesized or utilized by certain rumen microorganisms, which may in turn affect the rumen microbiota and consequently rumen digestion. However, there is currently no research available on the impact of 5-ALA on rumen digestion, although previous studies suggest that it theoretically has a positive effect on ruminal digestibility. The physiological and biochemical characteristics of 5-ALA support this hypothesis. In this study, a gradient dosage of 5-ALA was supplemented into the substrate to evaluate its effects on in vitro gas production, fermentation parameters, and microbial diversity.

## 2. Materials and Methods

### 2.1. Experimental Design and In Vitro Procedure

The 5% 5-ALA additive solution used in this study was provided by Tianjin Bofield Technology Co., Ltd. (Tianjin City, China). An approximate 5-ALA dose range was determined after preliminary screening, and two lower doses (100 mg/kg and 500 mg/kg) and two higher doses (1000 mg/kg and 5000 mg/kg) were selected. Five levels of 5-ALA were supplemented into a total mixed ration as a fermentation substrate in increasing amounts: 0 (ALA0), 100 (ALA100), 500 (ALA500), 1000 (ALA1000), and 5000 (ALA5000) mg/kg on a dry matter (DM) basis. The total mixed ration was composed of 40% whole-plant corn silage and 60% commercial concentrate (3432, Shanxi Yijia Agriculture and Animal Husbandry Technology Co., Ltd., Pingyao, China). All the feed samples were dried at 65 °C for 48 h in a forced air oven to constant weight, ground in a hammer mill to pass through a 1 mm sieve, and stored in sampling bags for subsequent in vitro incubation.

In vitro incubation was carried out according to the procedure of Menke et al. [14]. Rumen inoculum was collected from 3 Jinnan cattle fitted with permanent rumen fistulas before morning feeding, strained through four layers of cheesecloth into a vacuum bottle, and transported immediately to the laboratory of the Institute of Shanxi Animal Husbandry and Veterinary Medicine of Shanxi Agricultural University. The rumen inoculum was mixed with a buffer solution in a 1:2 (*v*/*v*) ratio while continuously flushing with CO_2_ according to Menke and Steingass [14]. The mixed artificial rumen culture solution (~30 mL) was dispensed into 100 mL glass syringes, in which the total mixed ration samples of about 220 mg (air-dry matter basis) were weighed and placed in advance and kept in a 39 °C incubator. Subsequently, the 5-ALA additive dilution solution was accurately measured and injected into corresponding syringes through the rubber tube using a 1 mL medical syringe. A total of 26 glass syringes, 4 syringes for each of 5 supplemental levels with 6 syringes as blanks (i.e., artificial rumen culture solution only), were incubated at 39 °C and 60 r/min for 72 h.

Two sets of in vitro tests were performed in this study. One set was used to assess gas production (GP), fermentation characteristics, and bacterial community profiles, while the other set was conducted to measure dry matter degradation (DMD).

### 2.2. Data Collection, Sampling, and Analysis

The volume of GP was recorded manually at time-points of 0, 1, 2, 4, 6, 8, 10, 12, 16, 20, 24, 28, 32, 36, 40, 44, 48, 60, and 72 h of incubation. At the end of cultivation (i.e., 72 h), the fermentation mixture was dispensed into two 50 mL centrifuge tubes. One of which was immediately frozen in liquid nitrogen and stored at −80 °C for microbial diversity analysis. The other was measured for the final pH using a portable pH meter (S-25, Shanghai INESA Scientific Instrument Co., Ltd., Shanghai, China) and then centrifugated at 8000× *g* and 4 °C for 15 min and passed through a 0.45 μm filter membrane to obtain the supernatant for the determination of volatile fatty acids (VFA) profiles and ammonia-nitrogen (NH_3_-N). The VFA concentration was determined using HPLC equipped with a Hi-Plex H ligand-exchange column (300 mm × 7.7 mm, 8 μm) as stated by Zhang et al. [15]. The NH_3_-N concentration was colorimetrically measured based on the method described by Broderick and Kang [16].

Regarding the DMD determination, the entire fermentation mixture was transferred into a 50 mL centrifuge tube and then centrifugated, as described above, to obtain the non-fermented residues. The precipitate was rinsed with distilled water and centrifuged again, and then was dried at 105 °C for constant weight to estimate the disappearance of DM by calculating the loss in weight after drying.

### 2.3. Bacterial Amplicon Sequencing and Data Processing

Whole genomic DNA was extracted from the fermentation mixture samples using the E.Z.N.A.^®^ soil DNA Kit (Omega Bio-tek, Norcross, GA, USA) according to the manufacturer’s instructions. The purity, concentration, and integrity of the extracted DNA were determined with a NanoDrop 2000 UV-vis spectrophotometer (Thermo Scientific, Wilmington, DE, USA) and 1% agarose gel electrophoresis, respectively. The verified DNA was used as a template to amplify the hypervariable region V3-V4 of the bacterial 16S rRNA gene with the primer pairs 338F (5′-ACTCCTACGGGAGGCAGCAG-3′) and 806R (5′-GGACTACHVGGGTWTCTAAT-3′) via an ABI GeneAmp^®^ 9700 PCR thermocycler (ABI, Carlsbad, CA, USA). The PCR reaction mixture consisted of 5 × *TransStart* FastPfu buffer 4 μL, 2.5 mM dNTPs 2 μL, forward primer (5 μM) 0.8 μL, reverse primer (5 μM) 0.8 μL, *TransStart* FastPfu polymerase 0.4 μL, bovine serum albumin (BSA) 0.2 μL, template DNA 10 ng, and finally ddH_2_O up to 20 μL. The PCR amplification process was carried out as follows: initial denaturation at 95 °C for 3 min, followed by 27 cycles (denaturation at 95 °C for 30 s, annealing at 55 °C for 30 s, and extension at 72 °C for 45 s) and single stable extension at 72 °C for 10 min and was finally ended at 4 °C. The PCR products underwent enrichment via 2% agarose gel electrophoresis and were then purified using the AxyPrep DNA Gel Extraction Kit (Axygen Biosciences, Union City, CA, USA) following the manufacturer’s instructions. The purified products were then quantified using a Quantus™ Fluorometer (Promega, Madison, WI, USA). Only the amplicon samples that had the correct PCR bands and appropriate concentrations were used for subsequent library construction and sequencing. The sequencing library was established by the use of the TruSeq^TM^ DNA Library Prep Kit (Illumina, San Diego, CA, USA) following the merchandise instructions. The resulting amplicons were then pooled in equimolar amounts and subjected to paired-end sequencing on an Illumina PE300 platform (Illumina, San Diego, CA, USA) according to the standard protocols by Majorbio Bio-Pharm Technology Co., Ltd. (Shanghai, China).

Off-line sequencing data were processed and analyzed using the platform QIIME2 (version 2022.08) as described previously with modification [17,18]. Briefly, the barcode and primer sequences were removed from the raw paired reads via the trim-paired method integrated into the q2-cutadapt plugin [19]. The generated paired reads were denoised, merged, dereplicated, and chimera-filtered using the denoise-paired method integrated into the q2-dada2 plugin to obtain amplicon sequence variants (ASVs) and their frequency distribution tables [20]. This process was achieved by truncating forward and reverse reads at position 270 and 199 bases, respectively. Subsequently, the taxonomic classification of the ASVs was performed as described by Zhang et al. [18].

A phylogenetic tree was generated to analyze phylogenetic diversity (PD) using the align-to-tree-mafft-fasttree pipeline integrated into the q2-phylogeny plugin. Alpha rarefaction plots based on the Shannon index and Faith PD metrics were used to assess if sample richness has been fully observed. The sequence count of all samples was standardized by rarefying them to the same number of sequences (27,867 in this study) to comparably analyze the bacterial diversity among samples. The rarefied feature table and the phylogenetic tree were then used to compute alpha diversity indices, including the Shannon index, observed features, Faith PD, and Pielou evenness and beta diversity metrics, including Bray–Curtis and Jaccard distance, using the core-metrics-phylogenetic pipeline integrated into the q2-diversity plugin. Moreover, the microbial function was predicted using the q2-picrust2 plugin, and metabolic pathways were annotated using the Metcyc database.

### 2.4. Calculations and Statistical Analysis

To estimate the kinetic parameters of GP, the results from in vitro gas production were fitted using the NLIN Procedure of the SAS software (version 3.81) following the method described by France et al. [21] as follows:

GP = b (1 − e^−ct^)

where GP (mL/0.22 g) represents the volume of gas production per 0.22 g substrate at time t, b (mL) is the asymptotic gas production of 0.22 g substrate, and c (mL/h) is the rate of gas production per hour.

A general linear model considering the 5-ALA level as a fixed effect was applied for data from bacterial relative abundance, gas production, and fermentation parameters in the GLM Procedure of the SAS software as follows:

Y_i_ = μ + T_i_ + e_i_

where Y_i_ is the observation of the ith 5-ALA level; μ and e_i_ represent the general mean and random residual error, respectively; and T_i_ represents the effect of the 5-ALA level at the ith dosage. Differences in the least-squares means of the fixed effects were pairwise compared using Duncan’s test and declared significant at *p* < 0.05.

Regarding microbial diversity analysis, the differences in alpha diversity among different 5-ALA levels were evaluated with the Kruskal–Wallis test through the alpha-group-significance command from the q2-diversity plugin. Principal coordinate analysis (PCoA) and permutational multivariate analysis of variance (PERMANOVA) was applied to visualize bacterial community profile based on Bray–Curtis and Jaccard distances at the ASVs level using the qiime2R (version 0.99.6) package in R (version 4.3.1).

For the microbial composition analysis, we summarized the bacterial composition profiles at phylum and genus levels. At each taxonomic level, bacterial taxa with a relative abundance greater than 0.01% in at least one sample were considered as being identified, while those with a relative abundance greater than 0.01% and present in more than half of the samples were defined as being detected and used for downstream statistical analysis. Relative abundance of bacterial taxa and metabolic pathways were arcsine square root transformed and then compared among different 5-ALA levels using one-way analysis of variance in the GLM procedure as described above. The more stringent linear discriminant analysis (LDA) effect size (LEfSe) was performed to further identify differentially abundant bacterial taxa and metabolic pathways, as described by Segata et al. [22]. The bacterial taxa with *p* < 0.05 and LDA score > 3 and metabolic pathways with *p* < 0.05 and LDA score > 2 were considered to be significantly different.

Subsequently, spearman rank correlation analysis was performed using the psych package in R to investigate the relationship between gas production, fermentation parameters, and the relative abundance of detectable phyla and top 20 genera. A correlation was considered significant when *p* < 0.05, while a highly significant correlation was indicated by *p* < 0.01.

## 3. Results

### 3.1. In Vitro Gas Production Kinetics and Dry Matter Digestibility

**Figure 1:**
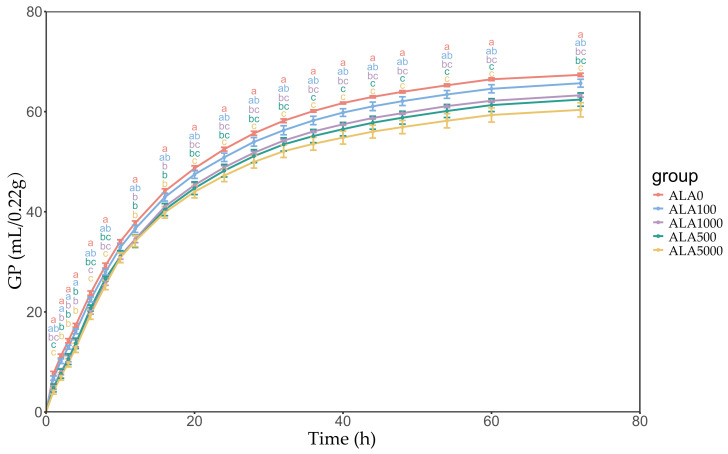
Gas production (GP) curves from in vitro rumen fermentation with 5-ALA levels. ALA0, ALA100, ALA500, ALA1000, and ALA5000 indicated that the content of 5-ALA in the fermentation substrate was 0, 100, 500, 1000, and 5000 mg/kg DM, respectively. Average ± standard error was used to indicate GP at each time point. At each timepoint, letters a, b, and c describe significant differences at *p* < 0.05.

Data concerning in vitro gas production kinetics parameters, DMD, and gas curves are presented in Table 1 and Figure 1. The addition levels of 5-ALA had a significant effect on the GP parameter b and DMD (*p* < 0.01) but did not affect the GP parameter c (*p* = 0.32). The asymptotic gas volume (b) decreased linearly and quadratically (*p* < 0.01), while DMD increased quadratically (*p* < 0.01) with 5-ALA levels.

### 3.2. In Vitro Fermentation Parameters

The in vitro fermentation parameters (pH, NH_3_-N, and VFA profiles) of 72 h incubation cultures with 5-ALA levels are summarized in Table 2. In general, the addition of 5-ALA significantly affected the final pH, the concentration of butyrate, isobutyrate, valerate, isovalerate, and NH_3_-N, and the ratio of acetate/propionate (A/P) (*p* < 0.05). However, it did not affect the concentration of acetate, propionate, and total VFA (*p* > 0.05). With the addition of 5-ALA, the final pH increased linearly and quadratically (*p* < 0.05) and propionate content increased linearly (*p* = 0.03), while the concentration of butyrate, isobutyrate, valerate, isovalerate, and NH_3_-N decreased linearly and quadratically (*p* < 0.05) and the ratio of acetate to propionate decreased quadratically (*p* < 0.01).

### 3.3. Sequencing Overview and Bacterial Diversity

In this study, a total of 1,457,870 pair-end reads (72,893 ± 9274; *n* = 20) were generated in the amplicon sequencing. After quality control, including primer removal, denoising, merging, and chimera-filtering, a total of 669,710 (33,486 ± 4358; *n* = 20) high-quality amplicon sequences were obtained, with 10,027 representative sequences (i.e., ASVs) averaging 416 ± 11 bases in length. The Good’s coverage of each sample was greater than 0.99, while the rarefaction curves based on the Shannon index and Faith PD for each sample level out as the sampling depth outnumbered 20,000 (Supplementary Figure S1), indicating that the sequencing analysis was deep enough to cover the entire bacterial community, assuring the representativeness of the sequencing data.

**Figure 2:**
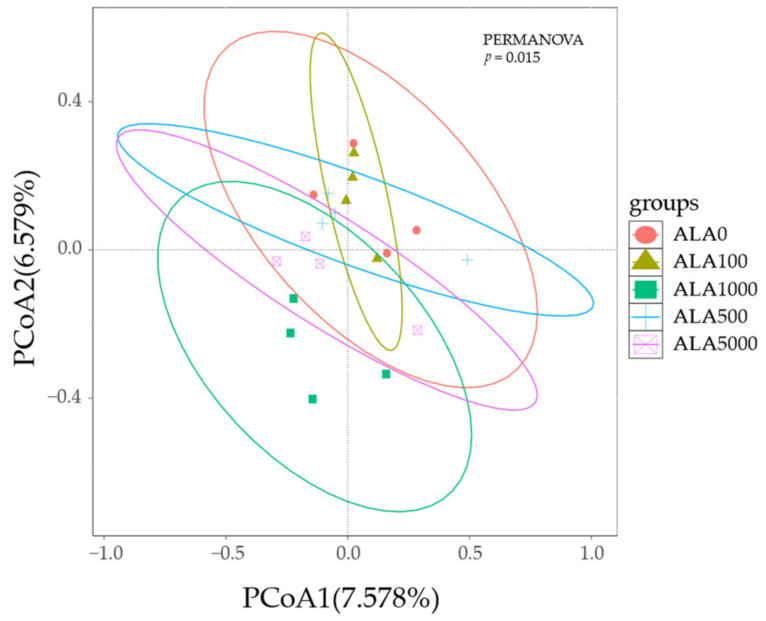
Dissimilarity of the bacterial profiles in the in vitro rumen cultures with 5-ALA levels. Distance between samples based on similarity in ASVs composition was calculated using Jaccard distance and visualized using PCoA plots. The impact of 5-ALA levels on the clustering pattern of bacterial communities was tested using PERMANOVA. The ovals in varied colors represent a 95% confidence interval of bacterial profiles at various 5-ALA levels. ALA0, ALA100, ALA500, ALA1000, and ALA5000 indicated that the content of 5-ALA in the fermentation substrate was 0, 100, 500, 1000, and 5000 mg/kg DM, respectively.

Variations in the alpha and beta diversity of bacterial communities in in vitro rumen cultures with 5-ALA levels were demonstrated in Table 3 and Figure 2, respectively. Results showed that the addition of 5-ALA did not significantly impact any alpha diversity indices of bacterial communities in in vitro rumen cultures (*p* > 0.05, Table 3). PCoA results indicated that the samples of each treatment group were individually clustered within a limited range, with the confidence ellipses of each treatment group tending to separate from each other (Figure 2). PERMNOVA further demonstrated that there were significant differences (*p <* 0.05) in the bacterial profiles among different 5-ALA treatment groups (*p* = 0.015), with the ALA1000 group showing statistical differences with other groups (*p <* 0.05) except for the ALA5000 group (*p =* 0.13).

**Figure 3:**
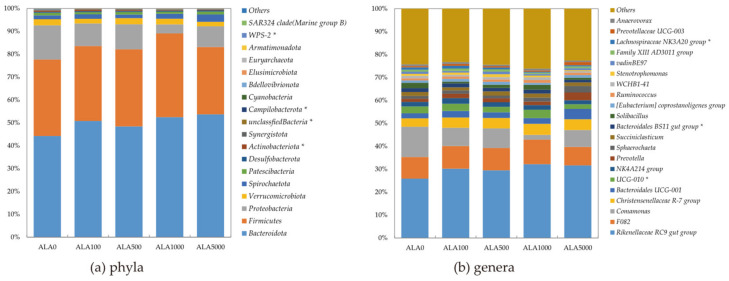
Bacterial composition at the phylum (**a**) and genus (**b**) level in the in vitro rumen cultures with 5-ALA levels. ALA0, ALA100, ALA500, ALA1000, and ALA5000 indicated that the content of 5-ALA in the fermentation substrate was 0, 100, 500, 1000, and 5000 mg/kg DM, respectively. The group Others included taxa with a relative abundance less than 0.01% or presented in less than half of the samples. Taxa name with asterisk (*) denotes significant 5-ALA dose effect (*p* < 0.05).

Bacterial composition at both the phylum and genus levels in the in vitro rumen cultures with 5-ALA levels were illustrated in Figure 3 and Supplementary Tables S1 and S2. Taxonomic annotation revealed a total of 23 and 380 bacterial taxa at the phylum and genus level as being identified, 18 and 173 of which were classified as being detected, respectively. The top six abundant phyla were *Bacteroidota, Firmicutes, Proteobacteria, Verrucomicrobiota, Spirochaetota, and Patescibacteria*, with an average relative abundance of 49.92, 33.19, 9.76, 2.35, 2.12, and 1.07% in the in vitro rumen cultures, respectively. At the genus level, the average relative abundance of 14 bacterial taxa was greater than 1%. The top three dominant genera were the *Rikenellaceae RC9 gut group* (29.9%), *F082* (9.57%), and *Comamonas* (7.84%).

**Figure 4:**
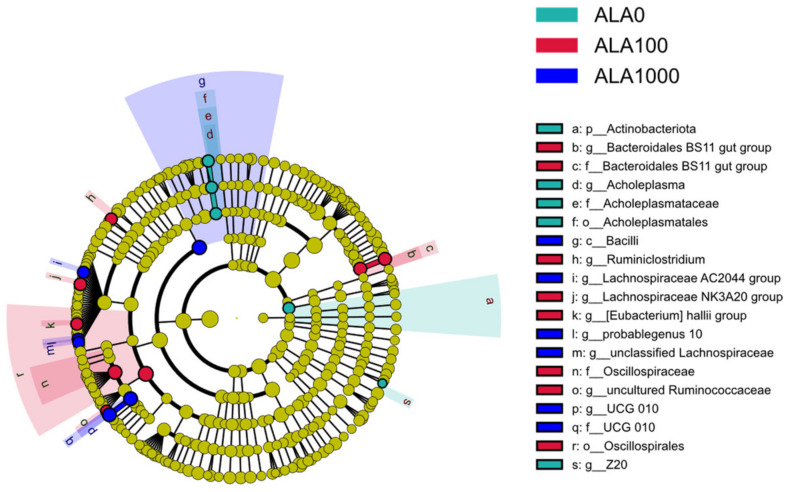
Differential bacterial taxa (*p* < 0.05, LDA score > 3) in in vitro rumen cultures with 5-ALA levels via LEfSe. ALA0, ALA100, and ALA1000 indicated that the content of 5-ALA in the fermentation substrate was 0, 100, and 1000 mg/kg DM, respectively.

LEfSe analysis revealed a significant difference in the relative abundance of 19 detected bacterial taxa among treatment groups (Figure 4). At the phylum level, *Actinobacteriota*, *Acholeplasma*, *Acholeplasmataceae*, *Acholeplasmataceae*, and *Z20* were significantly enriched in group ALA0 (*p* < 0.05, LDA score > 3). The relative abundance of five genera, *Ruminiclostridium*, the *Lachnospiraceae NK3A20 group*, the *Bacteroidales BS11 gut group*, *and* the *Eubacterium hallii group*, is highest in group ALA100 (*p* < 0.05, LDA score > 3). Group ALA1000 had the highest abundance of *Lachnospiraceae AC2044 group*, *UCG_010*, *probable genus 10*, and *unclassified Lachnospiraceae* (*p* < 0.05, LDA score > 3).

### 3.4. Correlation of Bacterial Taxa with In Vitro Gas Production and Fermentation Parameters

**Figure 5:**
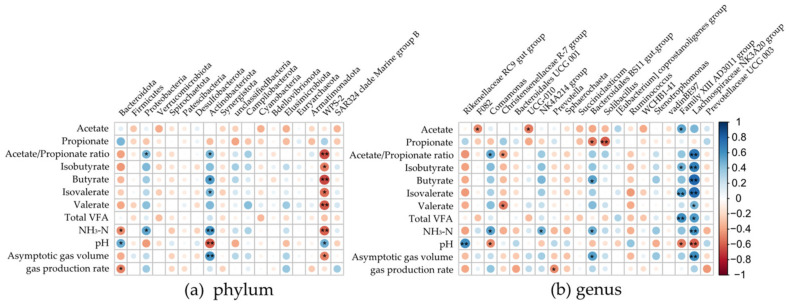
Correlation of gas production and fermentation parameters with bacterial abundance at phylum (**a**) and genus (**b**) levels. A single asterisk (*) in a box indicates a weaker correlation (*p* < 0.05), and a double asterisk (**) in a box indicates a strong correlation (*p* < 0.01). Total VFA = acetate + propionate + butyrate + isobutyrate + valerate + isovalerate.

The correlation between specific bacterial taxa and in vitro gas production and fermentation parameters are depicted in Figure 5. Results showed that at the phylum level (Figure 5a), *Bacteroidota* has a negative correlation with NH_3_-N content and gas parameter c (*p* < 0.05), but a positive correlation with the pH of the 72 h incubation fluid (*p* < 0.05). *Actinobacteriota* displayed a positive correlation with the ratio of A/P and the concentration of butyrate and isovalerate (*p* < 0.05). It also showed a highly positive correlation with NH_3_-N content and gas parameter b (*p* < 0.01) while displaying a highly negative correlation with pH (*p* < 0.01). *WPS-2* exhibited a strong negative correlation with the ratio of A/P and the concentration of butyrate, valerate, and NH_3_-N (*p* < 0.01) as well as a weaker negative correlation with b and the concentration of isobutyrate and isovalerate (*p* < 0.05) but a weaker positive correlation with pH (*p* = 0.04).

At the genus level (Figure 5b), the *Rikenellaceae RC9 gut group* showed a strong positive correlation with pH (*p* < 0.01). The *Bacteroidales BS11 gut group* was negatively correlated with the concentration of propionate (*p* = 0.02) but positively correlated with gas parameter b and the concentration of butyrate and NH_3_-N (*p* < 0.01). The *Family XIII AD3011 group* displayed a positive correlation with the concentration of acetate and isobutyrate (*p* < 0.05), a strong positive correlation with the concentration of isovalerate and total VFA (*p* < 0.01), and a negative correlation with pH (*p* = 0.03). Moreover, the *Lachnospiraceae NK3A20 group* was found to have a strong positive correlation with gas parameter b, the ratio of A/P, and the concentration of butyrate, isobutyrate, isovalerate, and NH_3_-N (*p* < 0.01). It also showed a positive correlation with the concentration of valerate and total VFA (*p* < 0.05) but a highly negative correlation with pH (*p* < 0.01).

### 3.5. Metabolic Pathways Predicted from Bacterial Communities in In Vitro Rumen Cultures with 5-ALA Levels

**Figure 6:**
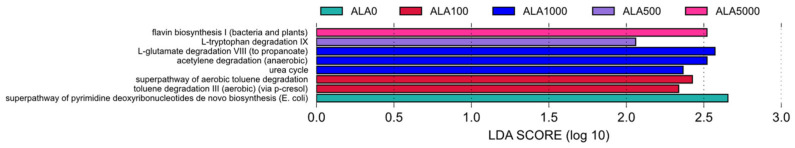
Differential metabolic pathways predicted from bacterial communities in in vitro rumen cultures with 5-ALA levels via LEfSe (*p* < 0.05, LDA score > 2). ALA0, ALA100, ALA500, ALA1000, and ALA5000 indicated that the content of 5-ALA in the fermentation substrate was 0, 100, 500, 1000, and 5000 mg/kg DM, respectively.

Differential metabolic pathways predicted from bacterial communities in in vitro rumen cultures with 5-ALA levels are demonstrated in Figure 6. LEfSe analysis identified a total of eight differential metabolic pathways (*p* < 0.05, LDA score > 2). The superpathway of pyrimidine deoxyribonucleotides de novo biosynthesis (*E. coli*), L-tryptophan degradation IX, and flavin biosynthesis I (bacteria and plants) were significantly enriched in group ALA0, ALA500, and ALA5000, respectively. Group ALA100 had the richest metabolic pathway for toluene degradation III (aerobic) (via p-cresol) and the superpathway of aerobic toluene degradation. Three metabolic pathways, including L-glutamate degradation VIII (to propanoate), acetylene degradation (anaerobic), and urea cycle, exhibited the highest abundance in group ALA1000.

## 4. Discussion

Nutrients in feed, such as carbohydrates and proteins, are degraded by rumen microorganisms to produce a large amount of VFAs, CH_4_, CO_2_, NH_3_, and a small amount of H_2_. As an indicator reflecting the degree of dietary degradation in the rumen, DMD has a specific correlation with the degradation rate of feed protein and neutral detergent fiber [23]. Studies suggested that gas production was positively correlated with DMD [24]. In the current study, however, the supplement of 5-ALA linearly and quadratically decreased the GP parameter b while it quadratically increased the DMD. These paradoxical results might be attributed to the fermentation type (i.e., the ratio of A/P). Previous studies have highlighted that propionic-type fermentation can compete with H_2_ with methanogens, reducing CH_4_ and H_2_ production [25]. Moreover, higher DMD may lead to higher feed intake in beef cattle. The yield of ammonia nitrogen decreased with the increase in 5-ALA addition, which was also an important reason for the decrease in gas content in this experiment.

Ruminal pH is a crucial factor modulating microorganisms’ growth in the rumen. The ruminal pH typically ranges from 5.5 to 7.5, and the optimal pH range for ruminal microbial fermentation ranges from 6.6 to 7.2 [26]. The pH range of fermentation cultures in this study was from 6.65 to 6.96, which did not adversely affect rumen microbial activity. The pH value of the in vitro culture is the balance between acids generated from feed fermentation and buffering capacity, which depends on the yield and composition of volatile fatty acids and the concentration of NH_3_-N. However, as the supplemental level of 5-ALA increased in this study, the pH increased linearly and quadratically, while the total VFA concentration did not change significantly, and the NH_3_-N concentration decreased linearly and quadratically. The reason for the decreased pH might be due to the decreased A/P. As is well known, propionic acid has a lower dissociation constant than acetic acid, resulting in a higher pH with a reduced A/P ratio, despite similar total VFA concentration.

Volatile fatty acids produced through carbohydrate fermentation, mainly acetic, propionic, and butyric acids, are the primary sources of energy and carbon for ruminants, providing approximately 70~80% of their energy requirements [27]. Acetic acid serves as the primary precursor for milk and body fat in ruminants, while propionic acid is principally utilized for gluconeogenesis in the liver [28]. Cobalamin, as a derivative of 5-ALA, is a crucial cofactor of methionine synthetase and methylmalonyl-CoA mutase, essential for propionic acid production by rumen microorganisms [29]. Increased supplementation of 5-ALA led to higher cobalamin synthesis by rumen microorganisms, resulting in increased propionic acid production, which might have caused a decrease in the A/P ratio in this study. Furthermore, the decreased A/P ratio indicates the transformation of the fermentation pattern to propionic-type fermentation. Propionic-type fermentation competes with methanogens for H_2_, thus reducing energy loss and improving energy efficiency [30]. Moreover, 85% to 90% butyric acid synthesizes *β*-hydroxybutyric acid to fuel the muscles and brain during a state of starvation [31]. The decrease in butyrate concentration might be linked to the reduced relative abundance of butyric acid fermentation bacteria in the fermentation cultures, such as the *Eubacterium hallii group*, *Bacteroidales BS11 gut group*, and *Lachnospiraceae NK3A20 group* [32,33]. The *Eubacterium hallii group* is a facultative anaerobic gram-negative bacterium that can synthesize butyric acid from glucose [34]. In this study, its relative abundance decreased with the 5-ALA supplement and was highly positively correlated (Spearman correlation coefficient = 0.66, *p* < 0.01) with the butyrate concentration in the fermentation cultures.

A linear and quadric decline in NH_3_-N was observed with the addition of 5-ALA in this study. NH_3_-N is the product of protein degradation by rumen microorganisms and is also used as a raw material for bacterial protein synthesis. Previous studies on both monogastric and ruminant animals have suggested that dietary 5-ALA supplementation could affect protein utilization [9,11,35]. The decrease in NH_3_-N concentration may be due to reduced dietary protein degradation or increased microbial protein synthesis. Considering the increased DMD, it seems more plausible that 5-ALA supplementation improved protein synthesis by rumen microorganisms. In addition, the decrease in the concentration of the isobutyrate, valerate, and isovalerate in the fermentation cultures also corresponded with the reduction in NH_3_-N concentration. Isoacids, such as isobutyric, valeric acid, and isovaleric acid, are the products of branched-chain amino acid degradation through deamination in the rumen [36]. They are also involved in the synthesis of branched-chain amino acids by microorganisms. Consequently, the change in NH_3_-N concentration should be consistent with the levels of isoacids.

In this study, 5-ALA supplementation did not significantly affect the alpha diversity of bacterial communities in the fermentation cultures. However, PCoA and PERMNOVA analyses based on the Jaccard distance revealed significant differences between ALA1000 and the other groups, but not with ALA5000. The Jacquard distance measures the difference between two sets, equal to one minus the Jacquard similarity index (the ratio of intersecting elements of two sets to the number of union elements). These findings suggested that 5-ALA had a noticeable impact on bacterial communities in in vitro fermentation cultures. In line with our findings, Chang et al. [11] suggested that 5-ALA could influence both alpha and beta diversity of the gut microbiome in pregnant sows, even with an additional amount of only 50 mg/kg.

Consistent with previous studies, the two most prominent bacterial phyla were *Bacteroidota* and *Firmicutes* in the fermentation cultures [37]. *Bacteroidetes* play a key role in breaking down dietary proteins and carbohydrates, as well as maintaining normal gastrointestinal tract functions [38]. *Firmicutes* are primarily involved in energy metabolism, cellulose degradation, and hemicellulose degradation [39]. At the genus level, the *Rikenellaceae RC9 gut group* had the highest relative abundance. This group belongs to the *Rikenellaceae* family, which was known for decomposing fibrous polysaccharides [40]. This could explain the positive correlation between the *Rikenellaceae RC9 gut group* and pH in this study.

*Actinobacteriota*, the *Bacteroidales BS11 gut group*, and the *Lachnospiraceae NK3A20 group* exhibited significant differences across 5-ALA levels and correlated with multiple gas production and fermentation parameters. Some bacteria belonging to *Actinobacteriota* are associated with cellulose degradation and intestinal homeostasis [41,42], while others are pathogenic. Interestingly, most of the detectable bacteria taxa in *Actinobacteriota* in this study, including *Eggerthellaceae*, *Corynebacterium*, *Olsenella*, and *Atopobium*, are associated with certain diseases [43,44,45,46]. Therefore, we speculate that adding 5-ALA may lead to a decline in pathogenic bacteria abundance in the rumen. In addition, *WPS-2* is an uncultured bacterial phylum that includes bacteria with diverse metabolic capabilities. It has been detected mainly in soil and the canine oral microbiome [47]. It is worth noting that even though we lowered the LDA threshold (*p* = 0.03, LDA score = 2.63), the most abundant *WPS-2* was still observed in the ALA1000 group. Furthermore, we observed a negative correlation between the abundance of *WPS-2* and the A/P ratio, as well as the concentration of butyrate, isoacids, and NH_3_-N in the fermentation cultures. These results suggested that the *WPS-2* could have a significant impact on ruminal fermentation, while the mechanism of 5-ALA’s effect on *WPS-2* remains unclear.

The *Bacteroidales BS11 gut group* has been known to specialize in fermenting various hemicellulosic monomers (xylose, fucose, mannose, and rhamnose) to produce acetate and butyrate [32]. In line with previous studies, we also observed a positive correlation between the abundance of the *Bacteroidales BS11 gut group* and the concentration of butyrate and a negative correlation with the concentration of propionate. The *Lachnospiraceae NK3A20 group* produced acetate, butyrate, H_2_, and formate from glucose [33]. This study also observed a strong positive correlation between the abundance of the *Lachnospiraceae NK3A20 group* and the A/P ratio, as well as butyrate concentration. In addition, the relative abundance of the *Bacteroidales BS11 gut group* and the *Lachnospiraceae NK3A20 group* decreased linearly with the 5-ALA levels. Furthermore, there was a linear increase in propionate concentration, indicating that their niche might be replaced by some propionic acid fermenter.

Based on the prediction results from Picrust2, we observed an increase in metabolic pathways related to crude protein utilization, L-glutamate degradation VIII (to propanoate), L-tryptophan degradation IX, and the urea cycle with 5-ALA levels. This change coincided with a decrease in NH_3_-N and isoacids concentration in fermentation cultures, suggesting that 5-ALA might enhance the utilization efficiency of protein by rumen microorganisms.

## 5. Conclusions

In summary, including 5-ALA in the diet could reduce the presence of ruminal bacteria that are involved in acetic and butyric acid fermentation, such as *Eubacterium hallii group*, the *Bacteroidales BS11 gut group*, and the *Lachnospiraceae NK3A20 group*. These bacteria’s ecological niches could be replaced by propionic acid fermenters, potentially shifting the fermentation pattern towards propionic-type fermentation. This change has the potential to improve energy utilization efficiency. Additionally, the addition of 5-ALA might enhance the protein utilization of ruminal bacteria by altering the bacterial community structure and improving metabolic pathways related to protein utilization, such as L-glutamate degradation VIII (to propanoate), L-tryptophan degradation IX, and the urea cycle. Moreover, it might even reduce the presence of some harmful bacteria in *Actinobacteriota*. It is worth noting that the positive effects were pronounced when 5-ALA was added in amounts exceeding 1000 mg/kg DM. A feeding experiment could be conducted to further verify the impact of 5-ALA supplementation on beef cattle.

## Figures and Tables

**Table 1:** Gas production kinetics parameters and dry matter digestibility (DMD) from in vitro rumen fermentation with 5-ALA levels.

Items	Groups ^1^					SEM ^2^	*p*		
	ALA0	ALA100	ALA500	ALA1000	ALA5000		Group	Linear	Quadric
b ^3^, mL	66.10 ^a^	64.46 ^ab^	62.70 ^bc^	61.54 ^bc^	59.58 ^c^	0.669	<0.01	<0.01	<0.01
c ^4^, mL/h	0.071	0.069	0.065	0.066	0.067	0.001	0.32	0.58	0.16
DMD, %	72.23 ^c^	72.77 ^bc^	73.80 ^bc^	75.59 ^a^	74.39 ^ab^	0.353	<0.01	0.10	<0.01

^a~c^ In each row, each mean value for group letters a, b, and c in superscripts describes significant differences at *p* < 0.05. ^1^ Group, ALA0, ALA100, ALA500, ALA1000, and ALA5000 indicated that the content of 5-ALA in the fermentation substrate was 0, 100, 500, 1000, and 5000 mg/kg DM, respectively. ^2^ SEM, standard error of mean. ^3^ Asymptotic gas production of 0.22 g DM substrate. ^4^ The rate of gas production per hour.

**Table 2:** Fermentation parameters of in vitro rumen cultures after 72 h incubation with 5-ALA levels.

Items	Groups ^1^					SEM ^2^	*p*		
	ALA0	ALA100	ALA500	ALA1000	ALA5000		Group	Linear	Quadric
pH	6.69 ^b^	6.70 ^b^	6.72 ^b^	6.89 ^a^	6.82 ^a^	0.021	<0.01	0.04	<0.01
Acetate, mmol/L	50.05	50.32	50.35	48.72	50.64	0.280	0.29	0.45	0.18
Propionate, mmol/L	15.28	15.58	15.71	15.74	16.06	0.106	0.17	0.03	0.55
Acetate/Propionate ratio	3.28 ^a^	3.23 ^ab^	3.20 ^ab^	3.09 ^c^	3.15 ^bc^	0.018	<0.01	0.07	<0.01
Butyrate, mmol/L	7.12 ^a^	6.94 ^a^	6.48 ^b^	5.95 ^c^	5.92 ^c^	0.129	<0.01	<0.01	<0.01
Isobutyrate, mmol/L	0.88 ^a^	0.88 ^a^	0.86 ^a^	0.79 ^b^	0.76 ^b^	0.013	<0.01	<0.01	<0.01
Valerate, mmol/L	1.13 ^a^	1.13 ^a^	1.06 ^ab^	1.02 ^b^	1.02 ^b^	0.015	0.01	0.02	<0.01
Isovalerate, mmol/L	1.78 ^a^	1.76 ^a^	1.72 ^ab^	1.61^b^	1.62 ^b^	0.023	0.02	0.02	<0.01
Total VFA ^3^, mmol/L	76.24	76.61	76.18	73.84	76.02	0.405	0.27	0.88	0.13
NH_3_-N, mg/dL	22.20 ^a^	21.67 ^ab^	20.48 ^b^	18.08 ^c^	16.67 ^c^	0.570	<0.01	<0.01	<0.01

^a~c^ In each row, each mean value for group letters a, b, and c in superscripts describes significant differences at *p* < 0.05. ^1^ Group, ALA0, ALA100, ALA500, ALA1000, and ALA5000 indicated that the content of 5-ALA in the fermentation substrate was 0, 100, 500, 1000, and 5000 mg/kg DM, respectively. ^2^ SEM, standard error of mean. ^3^ Total VFA = Acetate + Propionate + butyrate + isobutyrate + valerate + isovalerate.

**Table 3:** Alpha diversity of the bacterial community in in vitro rumen cultures with 5-ALA levels.

Items	Groups ^1^					SEM ^2^	*p*		
	ALA0	ALA100	ALA500	ALA1000	ALA5000		Group	Linear	Quadric
Observed features	1261	1269	1357	1238	1246	26.44	0.67	0.60	0.87
Shannon index	8.47	8.76	8.76	8.91	8.57	0.083	0.50	0.64	0.27
Pielou evenness	0.82	0.85	0.84	0.87	0.83	0.007	0.25	0.72	0.16
Faith PD	102.85	104.12	107.60	100.51	97.17	1.654	0.36	0.10	0.27
Simpson index	0.98	0.99	0.99	0.99	0.98	0.002	0.36	0.96	0.22

^1^ ALA0, ALA100, ALA500, ALA1000, and ALA5000 indicated that the content of 5-ALA in the fermentation substrate was 0, 100, 500, 1000, and 5000 mg/kg DM, respectively. ^2^ SEM, standard error of mean.

## Data Availability

The datasets generated for this study can be found in the Sequence Read Archive (SRA) of the National Center for Biotechnology Information (NCBI) under accession number PRJNA1091551.

## Supplementary Materials

The following supporting information can be downloaded at: https://www.mdpi.com/article/10.3390/microorganisms12091867/s1, Table S1: Relative abundance of bacterial community at the phylum level in 72 h rumen incubation fluid in vitro; Table S2: Relative abundance of the top 20 abundant bacterial taxa at the genus level in 72 h rumen incubation fluid in vitro. Figure S1: Alpha rarefaction plots based on Shannon index and Faith PD.
