# Supplementary material for: Effects of 5-Aminolevulinic Acid Supplementation on Gas Production, Fermentation Characteristics, and Bacterial Community Profiles In Vitro
Source: Microorganisms. 2024 Sep 9;12(9):1867. doi: 10.3390/microorganisms12091867 (PMC11433865; doi:10.3390/microorganisms12091867)
Supplement: Supplementary file 1 [file microorganisms-12-01867-s001.zip › microorganisms-3174847-supplementary.pdf]

microorganisms

Supplementary Material

Supplementary Table S1. Relative abundance of bacterial community at the phylum level in 72 h rumen incubation fluid in vitro, %

Item	Treatment					SEM	P
	ALA0	ALA100	ALA500	ALA1000	ALA5000		
Bacteroidota	44.24	50.82	48.40	52.45	53.68	1.361	0.19
Firmicutes	33.40	32.75	33.72	36.63	29.45	0.818	0.08
Proteobacteria	15.00	9.85	10.95	3.95	9.06	1.458	0.20
Verrucomicrobiota	2.61	2.05	2.68	2.48	1.95	0.211	0.89
Spirochaetota	1.68	1.97	1.54	2.17	3.24	0.407	0.88
Patescibacteria	1.14	0.92	1.13	0.91	1.25	0.070	0.53
Desulfobacterota	0.42	0.47	0.44	0.39	0.51	0.032	0.82
Actinobacteriota	0.54[a]	0.54[a]	0.43[ab]	0.25[b]	0.25[b]	0.044	0.04
Synergistota	0.25	0.19	0.27	0.26	0.23	0.029	0.95
unclassfiedBacteria	0.15[a]	0.12[a]	0.13[a]	0.13[a]	0.07[b]	0.009	0.04
Campilobacterota	0.20[a]	0.04[b]	0.04[b]	0.05[b]	0.05[b]	0.018	0.04
Cyanobacteria	0.06	0.06	0.05	0.09	0.05	0.011	0.92
Bdellovibrionota	0.08	0.04	0.05	0.06	0.05	0.008	0.75
Elusimicrobiota	0.07	0.07	0.04	0.07	0.02	0.008	0.11
Euryarchaeota	0.03	0.02	0.05	0.01	0.04	0.006	0.45
Armatimonadota	0.03	0.03	0.02	0.02	0.03	0.004	0.73
WPS-2	0.01[c]	0.01[c]	0.01[bc]	0.03[a]	0.03[ab]	0.003	0.02
SAR324 clade (Marine group B)	0.01	0.02	0.01	0.02	0.01	0.003	0.85
Others	0.08	0.02	0.04	0.02	0.03	0.008	0.06

Note: ALA0, ALA100, ALA500, ALA1000, and ALA5000 indicated that the content of 5-ALA in the fermentation substrate was 0, 100, 500, 1000, and 5000 mg/kg DM, respectively.
SEM, standard error of means.
[a-c] Means in the same row followed by different superscripts differ ($P < 0.05$). P values were calculated based on arcsine square root transformed relative abundances among different supplement dose using R.

Supplementary Table S2. Relative abundance of the top 20 abundant bacterial taxa at the genus level in 72 h rumen incubation fluid in vitro, %

Item	Treatment					SEM	*P*
	ALA0	ALA100	ALA500	ALA1000	ALA5000		
Rikenellaceae RC9 gut group	25.87	30.23	29.52	32.15	31.66	0.796	0.07
F082	9.43	9.89	9.72	10.75	8.05	0.334	0.12
Comamonas	13.21	7.96	8.57	2.07	7.38	1.451	0.18
Christensenellaceae R-7 group	3.66	4.45	4.54	4.82	4.69	0.194	0.35
Bacteroidales UCG-001	2.19	2.88	2.48	2.52	4.49	0.442	0.73
UCG-010	3.00[ab]	3.09[ab]	2.36[bc]	3.60[a]	2.06[c]	0.158	0.00
NK4A214 group	1.99	2.50	2.02	2.05	1.75	0.089	0.08
Prevotella	1.34	1.90	1.71	1.51	3.37	0.344	0.55
Sphaerochaeta	1.24	1.47	1.16	1.76	2.88	0.374	0.79
Succiniclasticum	1.71	1.34	1.86	1.75	1.53	0.173	0.94
Bacteroidales BS11 gut group	1.71[a]	1.72[a]	1.49[ab]	1.69[a]	1.17[b]	0.068	0.01
Solibacillus	2.33	0.79	1.40	2.13	0.85	0.275	0.22
[Eubacterium] coprostanoligenes group	1.03	1.16	1.29	0.83	1.13	0.067	0.24
Ruminococcus	0.87	1.02	1.03	1.25	1.10	0.054	0.29
WCHB1-41	1.10	0.75	0.97	1.21	0.65	0.124	0.76
Stenotrophomonas	0.88	0.98	1.30	0.75	0.43	0.129	0.19
vadinBE97	0.95	0.70	1.10	0.68	0.87	0.089	0.69
Family XIII AD3011 group	0.86	1.07	1.02	0.57	0.76	0.069	0.10
Lachnospiraceae NK3A20 group	1.05[a]	1.18[a]	0.77[ab]	0.40[b]	0.49[b]	0.104	0.02
Prevotellaceae UCG-003	0.54	0.80	0.57	0.53	1.45	0.137	0.22

Note: ALA0, ALA100, ALA500, ALA1000, and ALA5000 indicated that the content of 5-ALA in the fermentation substrate was 0, 100, 500, 1000, and 5000 mg/kg DM, respectively.
SEM, standard error of means.
[a-c] Means in the same row followed by different superscripts differ ($P < 0.05$). P values were calculated based on arcsine square root transformed relative abundances among different supplement dose using R.

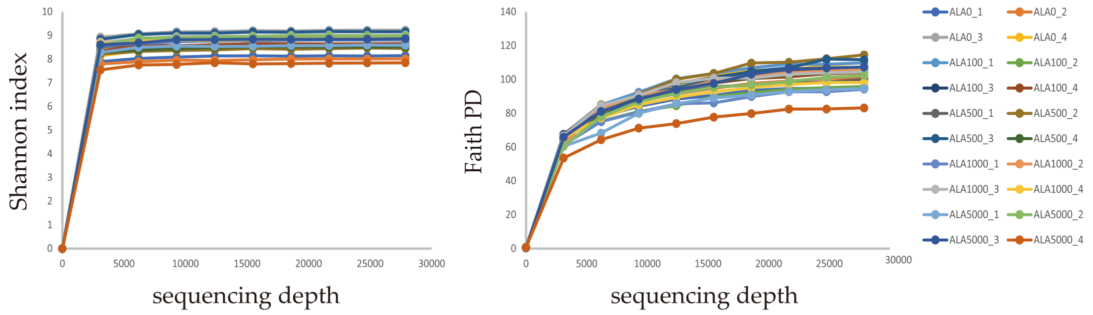

Supplementary Figure S1. Alpha rarefaction plots based on Shannon index and Faith PD, ALA0, ALA100, ALA500, ALA1000, and ALA5000 indicated that the content of 5-ALA in the fermentation substrate was 0, 100, 500, 1000, and 5000 mg/kg DM, respectively.
